# *Interleukin-6* Receptor rs7529229 T/C Polymorphism Is Associated with Left Main Coronary Artery Disease Phenotype in a Chinese Population

**DOI:** 10.3390/ijms15045623

**Published:** 2014-04-02

**Authors:** Feng He, Xiao Teng, Haiyong Gu, Hanning Liu, Zhou Zhou, Yan Zhao, Shengshou Hu, Zhe Zheng

**Affiliations:** 1 State Key Laboratory of Cardiovascular Disease, Fuwai Hospital, National Center for Cardiovascular Diseases, Chinese Academy of Medical Sciences and Peking Union Medical College, Beijing 100037, China; E-Mails: drhefeng@gmail.com (F.H.); tengxiao7@163.com (X.T.); zsylhn@gmail.com (H.L.); contyzhou@msn.com (Z.Z.); zhaoyan19791025@aliyun.com (Y.Z.); 2 Department of Cardiothoracic Surgery, Affiliated People’s Hospital of Jiangsu University, Zhenjiang 212002, China; E-Mail: haiyong_gu@hotmail.com

**Keywords:** *IL-6R*, polymorphisms, left main artery, coronary artery disease, mass spectrometry

## Abstract

Left main coronary artery disease (LMCAD) is a particular severe phenotype of coronary artery disease (CAD) and heritability. Interleukin (IL) may play important roles in the pathogenesis of CAD. Although several single nucleotide polymorphisms (SNPs) identified in *IL* related genes have been evaluated for their roles in inflammatory diseases and CAD predisposition, the investigations between genetic variants and CAD phenotype are limited. We hypothesized that some of these gene SNPs may contribute to LMCAD phenotype susceptibility compared with more peripheral coronary artery disease (MPCAD). In a hospital-based case-only study, we studied *IL-1A* rs1800587 C/T, *IL-1B* rs16944 G/A, *IL-6* rs1800796 C/G, *IL-6R* rs7529229 T/C, *IL-8* rs4073 T/A, *IL-10* rs1800872 A/C, and *IL-10* rs1800896 A/G SNPs in 402 LMCAD patients and 804 MPCAD patients in a Chinese population. Genotyping was done using matrix-assisted laser desorption/ionization time-of-flight mass spectrometry (MALDI-TOF MS) and ligation detection reaction (LDR) method. When the *IL-6R* rs7529229 TT homozygote genotype was used as the reference group, the CC or TC/CC genotypes were associated with the increased risk for LMCAD (CC *vs.* TT, adjusted odds ratio(OR) = 1.46, 95% confidence interval (CI) = 1.02–2.11, *p* = 0.042; CC + TC *vs*. TT, adjusted OR = 1.31, 95% CI = 1.02–1.69, *p* = 0.037). None of the other six SNPs achieved any significant differences between LMCAD and MPCAD. The present study suggests that *IL-6R* rs7529229 T/C functional SNP may contribute to the risk of LMCAD in a Chinese population. However, our results were limited. Validation by a larger study from a more diverse ethnic population is needed.

## 1. Introduction

Coronary artery disease (CAD) is the leading cause of mortality and morbidity in many countries including China [1,2]. Multifaceted phenotypes of CAD (number of involved vessels, location of lesions, severity of diameter narrowing, and morphology of lesions) have different mechanisms. Left main coronary artery disease (LMCAD) is a particular severe phenotype of CAD because it is associated with higher risk of fatal cardiovascular events [3]. Thus, any information in detecting the asymptomatic relatives of these patients can be useful for primary prevention.

LMCAD was heritability and frequently shared by siblings with CAD [4]. Fischer *et al.* [5] reported a stronger genetic component in LMCAD phenotype. However, Kolovou *et al.* [6] found no significant difference in cholesteryl ester transfer protein (CETP) allele frequency or genotype distribution among LMCAD and more peripheral coronary artery disease (MPCAD) patients. Thus, more investigations are needed in detecting association between genetic variants and CAD phenotype.

Inflammation is important in the initiation, progression, and clinical outcome of CAD [7,8]. Interleukin (IL) plays a key role in the inflammatory response, immune regulation and development of CAD. IL-1 family (including IL-1A, IL-1B *et al.*) plays a major role in inflammation by affecting antigen recognition patterns and lymphocyte function [9]. IL-6 is a multi-functional cytokine involved in various contradictory processes, a high circulating concentration of IL-6 is associated with increased risk of CAD [10]. IL-6 binds to IL-6R and then activates an intracellular signaling cascade leading to the inflammatory response [11]. IL-8 plays a key role in the development of atherosclerotic plaques [12]. IL-10 has anti-inflammatory and immunosuppressive effects by decreasing the production of pro-inflammatory mediators, exerts important protective effects on atherosclerotic lesion development [13].

Recently, a link between CAD genetic susceptibility and the response to inflammatory signaling has been established [8,14]. Growing evidence for heritability of pro-inflammatory state suggests that individual genetic background also modulate the development of CAD and its magnitude [15,16]. Despite well-described associations between inflammatory-related genetic variation and susceptibility to CAD, there is a paucity of data regarding to the phenotype of CAD.

The identification of novel genetic variants for use in assessing early risk of LMCAD have potentially important clinical implications, such as identifying high-risk individuals and adapting therapeutic management to the individual’s genetic make-up. Single nucleotide polymorphisms (SNPs) strongly influence the plasma levels and biological activity of the corresponding proteins. On the basis of the biological and pathologic significance of IL, we performed a genetic association study on the SNPs of *IL-1A*, *IL-1B*, *IL-6*, *IL-6R*, *IL-8* and *IL-10*.

The objective of this investigation was to evaluate the association between *IL* SNPs and LMCAD susceptibility compare with MPCAD in a hospital-based case-only study. We performed genotyping and analyses for the seven SNPs in a cohort of 1206 CAD patients (402 LMCAD patients and 804 MPCAD patients) in a Chinese population.

## 2. Results and Discussion

### 2.1. Characteristics of the Study Population

The demographic and clinical characteristics of all subjects are summarized in Table 1. Among 1206 DNA samples, the seven SNPs were successful ranging from 98.59% to 99.92% (Table 2). The mean age for LMCAD patients (62.24 ± 8.66) is significantly higher than for MPCAD patients (60.14 ± 8.96), *p* < 0.001 (Table 1). There are no significant difference for sex and mean body mass index (BMI), family history of CAD, previous smoker, hypertension, hyperlipidemia, diabetes mellitus, mean ejection fraction, circumflex branch of left coronary artery system, right coronary artery system, off-pump coronary artery bypass grafting (OPCAB) or conventional coronary artery bypass grafting (cCABG) for LMCAD patients and MPCAD patients (Table 1). LMCAD patients are more likely to have three disease territories than MPCAD patients (93.3% *vs.* 87.7%, *p* = 0.003) (Table 1).

### 2.2. Associations between the Seven SNPs and Risk of LMCAD and MPCAD

The genotype frequencies of the *IL-6R* rs7529229 T/C polymorphism were 34.1% (TT), 49.0% (TC) and 16.9% (CC) in LMCAD patients, and 40.4% (TT), 45.9% (TC) and 13.7% (CC) in MPCAD patients (*p* = 0.027) (Table 3). When the *IL-6R* rs7529229 TT homozygote genotype was used as the reference group, the CC genotype was associated with the increased risk for LMCAD (CC *vs.* TT, adjusted OR = 1.46, 95% CI = 1.02–2.11, *p* = 0.042). The TC genotype was not associated with the risk for LMCAD (TC *vs.* TT, adjusted OR = 1.26, 95% CI = 0.97–1.65, *p* = 0.088). In the dominant model, when the *IL-6R* rs7529229 TT genotype was used as the reference group, the TC/CC genotypes were associated with the increased risk for LMCAD (TC/CC *vs.* TT, adjusted OR = 1.31, 95% CI = 1.02–1.69, *p* = 0.037). The polymorphism was not associated with the risk for LMCAD in recessive genetic models (CC *vs.* TT/TC, adjusted OR = 1.29, 95% CI = 0.92–1.79, *p* = 0.140).

None of the other six polymorphisms achieved a significant difference in the genotype distributions between cases and controls. The six polymorphisms were not associated with the risk for LMCAD/MPCAD both in homozygote comparison, heterogeneity comparison, dominant genetic model and recessive genetic models (Table 3).

## 3. Experimental Section

### 3.1. Ethical Approval of the Study Protocol

The study protocol was approved by the Review Board of Peking Union Medical College (Beijing, China). We have complied with the World Medical Association Declaration of Helsinki regarding ethical conduct of research involving human subjects and/or animals. All patients provided written informed consent to be involved in the study.

### 3.2. Study Subjects

This study involved 1206 patients with CAD for the purpose undergoing coronary artery bypass grafting (CABG). Patients were consecutively recruited from the Cardiovascular Institute and Fuwai Hospital, Chinese Academy of Medical Sciences and Peking Union Medical College (Beijing, China) between December 2007 and December 2008. All patients were diagnosed using angiography which was scored systematically by an experienced interventional cardiologist. CAD patients were defined as having angiographic coronary stenosis of at least 50% lumen reduction. LMCAD was defined as at least 50% stenosis by visual assessment in the left main (LM) vessel, including ostial stenosis. While lesions compromising the lumen by ≥50% further from LM were defined as MPCAD [6,17].

All subjects were genetically unrelated ethnic Han Chinese. All data were collected by trained clinical research staff and were subsequently double entered into computer databases. Baseline information on personal and clinical characteristics was complete for all 1206 patients involved in the study.

### 3.3. Candidate Genes and SNPs Selection

Six candidate genes (*IL-1A*, *IL-1B*, *IL-6*, *IL-6R*, *IL-8* and *IL-10*) involved in the pathogenesis of inflammation and CAD were selected a priori based on previous transcription profiling in humans, pathway analysis, association studies reported in the literature, and expert opinion [18,19]. Seven typical SNPs, *IL-1A* rs1800587 C/T, *IL-1B* rs16944 G/A, *IL-6* rs1800796 C/G, *IL-6R* rs7529229 T/C, *IL-8* rs4073 T/A, *IL-10* rs1800872 A/C, and *IL-10* rs1800896 A/G, were subsequently selected in these candidate genes, based on function importance [20,21].

### 3.4. Isolation of DNA and Genotyping by Matrix-Assisted Laser Desorption/Ionization Time-of-Flight Mass Spectrometry (MALDI-TOF MS) and Ligation Detection Reaction (LDR)

Blood samples were collected using vacutainers and transferred to test tubes containing ethylenediamine tetra-acetic acid (EDTA). Genomic DNA was isolated from the lymphocytes of whole blood samples using the Wizard Genomic DNA Purification Kit (Promega, Madison, WI, USA). For quality control, all sample DNAs were conducted polymerase chain reaction (PCR), analyzed on a 3% agarose gel and visualized by ethidium bromide staining. *IL-1A* rs1800587 C/T, *IL-1B* rs16944 G/A, *IL-6* rs1800796 C/G, *IL-10* rs1800872 A/C and *IL-10* rs1800896 A/G genotyping was done by MALDI-TOF MS support from CapitalBio Corporation (Beijing, China), using the MassARRAY system (Sequenom, San Diego, CA, USA) as previously described [22]. LMCAD and MPCAD at a proportion of ≈1:2 were assayed. Completed genotyping reactions were spotted onto a 384-well spectroCHIP (Sequenom) using a MassARRAY Nanodispenser (Sequenom), and analyzed by MALDI-TOF-MS (Sequenom, San Diego, CA, USA). Genotype calling was done in real time with MassARRAY RT software (version 3.1; Sequenom), and analyzed using MassARRAY Typer software (version 4.0; Sequenom). For *IL-6R* rs7529229 T/C and *IL-8* rs4073 T/A, genotyping study was performed using the LDR method [23,24], with technical support from Shanghai Biowing Applied Biotechnology Company (Shanghai, China).

### 3.5. Statistical Analyses

Differences in demographics, variables, and genotypes of the seven SNPs were evaluated using a chi-squared test. The associations between the seven SNPs and risk of CAD phenotype were estimated by computing odds ratios (ORs) and 95% confidence intervals (CIs) using logistic regression analyses, and by using crude ORs and adjusted ORs. Statistical differences with *p* < 0.05 were considered significant, and all statistical analyses were done with SAS software (version 9.1.3; SAS Institute, Cary, NC, USA).

## 4. Conclusions

In this study, we found that the *IL-6R* rs7529229 T/C polymorphism may increase the risk of LMCAD compared with MPCAD. The CC genotype is associated with higher risk of LMCAD phenotype. None of the other six polymorphisms showed any overall predisposition to LMCAD phenotype susceptibility. To the best of our knowledge, this study provides the first evidence linking variation in the *IL-6R* gene and LMCAD phenotype risk in a Chinese population.

This finding is supported by studies displaying a higher heritability of LMCAD. Capodanno *et al.* [25] investigated the epidemiology and the clinical impact of different anatomical phenotypes of the LM coronary artery, and Iwasaki *et al.* [26] investigated the distribution of coronary atherosclerosis in patients with CAD. Their findings suggested that LM phenotypes are more likely to present with atherosclerotic disease and significant stenosis and are particularly heritable [25,26].

Our results showed that *IL-6R* rs7529229 CC genotype as well as C allele was more prevalent in the LMCAD group than in the MPCAD control group. *IL-6R* rs7529229 T/C polymorphism was associated with increased risk of LMCAD, which may lead to LMCAD by enhancing inflammation. However, it was not found to be statistically significant in other six candidate gene variants.

IL-6 is an important pleiotropic cytokine that has a broad range of humoral and cellular immune properties relating to inflammation, tissue injury and contributes to the clinical evolution of CAD [27]. IL-6 exerts its biological activities through the IL-6R. The *IL-6R* gene is located on human chromosome 1q21, a region that previous studies have reported to be linked to metabolic syndrome, type 2 diabetes and atrial fibrillation [28–30]. The human *IL-6R* gene is highly polymorphic and there is considerable variation in its expression between individuals. A number of SNPs have been reported and genetic variants in *IL-6R* gene are associated with several different kinds of diseases, including CAD [31]. *IL-6R* rs7529229, in which the polymorphism is localized to a functional domain of the receptor protein, is a T/C variation in the *IL-6R* gene (intronic) on human chromosome 1. In 40 studies including up to 133449 individuals, mendelian randomization analyses revealed that *IL-6R* rs7529229 T/C marking a non-synonymous *IL-6R* variant (rs8192284; p.Asp358Ala), was associated with increased circulating log IL-6 concentration as well as reduced C-reactive protein and fibrinogen concentrations [32]. This suggests that *IL-6R* rs7529229 variant is strongly associated with CAD and cardiovascular events.

In our study, Using Power and Sample Size Calculation (PS, version 3.0, 2009), considering *IL-6R* rs7529229 T/C mutant alleles in the LMCAD group, OR, LMCAD samples and MPCAD samples, the power of our analysis (α = 0.05) was 0.857 in 396 LMCAD cases and 793 MPCAD cases with adjusted OR = 1.46. This suggests that SNP of *IL-6R* gene seems to also have a causal role in development of LMCAD. Thus, the findings indicate that *IL-6R* rs7529229 polymorphism may have potential importance in screening individuals at high risk for developing LMCAD, and targeting of IL-6R could provide a novel therapeutic approach for the prevention of LMCAD.

Several limitations of the present study need to be addressed. First, this was a hospital-based study; selection bias was unavoidable; Second, the polymorphisms we investigated, based on their functional considerations, may not offer a comprehensive view of the genetic variability. Further fine-mapping analysis of *IL* genes or high-density whole genome genetic analyses evaluating different CAD phenotypes might give further insights to the pathophysiologic mechanisms underlying LMCAD; Third, a single case–case study is not sufficient to fully interpret the relationship between *IL-6R* rs7529229 T/C polymorphism and susceptibility to LMCAD because of the relatively small number of patients with LMCAD. Replication studies with larger numbers of subjects are necessary to confirm our findings; Finally, we did not evaluate plasma IL-6 levels and the function of *IL-6R* rs7529229 T/C, which restricted our analyses.

Despite the limitations, the present study provided strong evidence that *IL-6R* rs7529229 T/C functional polymorphism may contribute to the risk of LMCAD. However, our results were obtained from a moderate-sized sample, and therefore this is a preliminary conclusion. Validation by a larger study from a more diverse ethnic population is needed to confirm these findings.

## Figures and Tables

**Table 1.** Patient Demographics and Risk Factors with coronary artery disease (CAD) (Left main coronary artery disease (LMCAD) and more peripheral coronary artery disease (MPCAD)).

Variable	LMCAD b (*n* = 402)	MPCAD c (*n* = 804)	*p*-Value	All CAD (*n* = 1206)
Mean age, *y*	62.24 (±8.66)	60.14 (±8.96)	**<0.001**	60.84 (±8.91)
Woman, %	67 (16.7)	170 (21.1)	0.065	237 (19.7)
Mean BMI a, kg/m^2^	25.55 (±3.16)	25.75 (±3.13)	0.299	25.68 (±3.14)
Family history of CAD, %	21 (5.2)	32 (4.0)	0.323	53 (4.4)
Previous smoker, %	211 (52.5)	422 (52.5)	1.000	633 (52.5)
Hypertension, %	261 (64.9)	530 (65.9)	0.732	791 (65.6)
Hyperlipidemia, %	275 (68.4)	573 (71.3)	0.290	848 (70.4)
Diabetes mellitus, %	125 (31.1)	271 (33.7)	0.363	396 (32.8)
Mean ejection fraction, %	60.25 (±7.96)	59.55 (±8.76)	0.177	59.78 (±8.50)
Disease territories, %				
1–2	27 (6.7)	99 (12.3)	**0.003**	126 (10.4)
3	375 (93.3)	705 (87.7)	**0.003**	1080 (89.6)
LMCAD, %	402 (100)	0 (0)	—	402 (33.3)
Anterior descending artery system, %	386 (96.0)	793 (98.6)	**0.004**	1179 (97.8)
Circumflex branch of left coronary artery system, %	373 (92.8)	744 (92.5)	0.876	1117 (92.6)
Right coronary artery system, %	374 (93.0)	751 (93.4)	0.807	1125 (93.3)
OPCAB/cCABG d	209/193	438/366	0.414	647/559

a BMI: body mass index;

b LMCAD: left main coronary artery disease;

c MPCAD: more peripheral coronary artery disease;

Bold values are statistically significant (*p* < 0.05);

d OPCAB: off-pump coronary artery bypass grafting; cCABG: conventional coronary artery bypass grafting.

**Table 2.** Primary information for seven genotyped single nucleotide polymorphisms (SNPs).

Genotyped SNPs	Chr a	Regulome DB Score b	TFBS c	Splicing (ESE or ESS)	Location	MAF d for Chinese in Database	% Genotyping Value
*IL-1A*: rs1800587 C/T	2	5	Y	Y	5′ UTR	0.073	99.92
*IL-1B*: rs16944 G/A	2	1f	Y	—	5′ near gene	0.453	99.83
*IL-6*: rs1800796 C/G	7	4	Y	—	ncRNA	0.233	99.75
*IL-6R*: rs7529229 T/C	1	2b	—	—	intron	0.442	98.59
*IL-8*: rs4073 T/A	4	2b	Y	—	5′ near gene	0.389	98.84
*IL-10*: rs1800872 A/C	1	5	Y	—	5′ near gene	0.238	99.83
*IL-10*: rs1800896 A/G	1	6	Y	—	5′ near gene	0.059	99.83

a Chr, chromosome;

b http://www.regulomedb.org/;

c TFBS: Transcription Factor Binding Site;

d MAF: minor allele frequency.

**Table 3.** Main effects of SNPs on LMCAD risk.

Genotyped SNPs	Genotyping (AA/AB/BB) a		AB *vs.* AA	BB *vs.* AA	BB *vs.* (AA + AB)	(BB + AB) *vs.* AA	*p* Trend
	LMCAD (*n* = 402)	MPCAD (*n* = 804)	Adjusted OR (95% CI); *p*-Value	Adjusted OR (95% CI); *p*-Value	Adjusted OR (95% CI); *p*-Value	Adjusted OR (95% CI); *p*-Value	
*IL-1A*: rs1800587 C/T	331/67/3	645/145/14	0.91 (0.66–1.26); 0.570	0.42 (0.12–1.42); 0.175	0.43 (0.12–1.50); 0.183	0.87 (0.64–1.19); 0.374	0.314
*IL-1B*: rs16944 G/A	104/203/95	212/397/193	1.04 (0.78–1.39); 0.794	1.01 (0.72–1.42); 0.974	0.98 (0.74–1.30); 0.890	1.03 (0.78–1.35); 0.841	0.948
*IL-6*: rs1800796 C/G	182/174/46	358/348/95	0.97 (0.75–1.25); 0.797	0.96 (0.65–1.43); 0.856	0.98 (0.67–1.43); 0.916	0.97 (0.76–1.23); 0.782	0.970
*IL-6R*: rs7529229 T/C	135/194/67	320/364/109	1.26 (0.97–1.65); 0.088	**1.46 (1.02–2.11); 0.042**	1.29 (0.92–1.79); 0.140	**1.31 (1.02–1.69); 0.037**	0.080
*IL-8*: rs4073 T/A	127/198/71	280/375/141	1.18 (0.90–1.56); 0.226	1.14 (0.80–1.62); 0.482	1.03 (0.75–1.41); 0.862	1.17 (0.91–1.52); 0.231	0.545
*IL-10*: rs1800872 A/C	155/197/49	336/371/96	1.15 (0.89–1.49); 0.277	1.08 (0.73–1.60); 0.716	1.00 (0.69–1.44); 0.982	1.14 (0.89–1.46); 0.305	0.558
*IL-10*: rs1800896 A/G	330/69/2	639/154/10	0.86 (0.63–1.18); 0.350	0.38 (0.08–1.76); 0.217	0.39 (0.09–1.81); 0.230	0.83 (0.61–1.13); 0.242	0.316

a AA/AB/BB means homozygote, heterozygote and mutated homozygote;

Bold values are statistically significant (*p* < 0.05); Adjusted for age and sex.

## References

[1] Roger V.L., Go A.S., Lloyd-Jones D.M., Benjamin E.J., Berry J.D., Borden W.B., Bravata D.M., Dai S., Ford E.S., Fox C.S. (2012). Heart disease and stroke statistics—2012 update: A report from the American Heart Association. Circulation.

[2] He J., Gu D., Wu X., Reynolds K., Duan X., Yao C., Wang J., Chen C.S., Chen J., Wildman R.P. (2005). Major causes of death among men and women in China. N. Engl. J. Med.

[3] Fajadet J., Chieffo A. (2012). Current management of left main coronary artery disease. Eur. Heart J.

[4] Fischer M., Mayer B., Baessler A., Riegger G., Erdmann J., Hengstenberg C., Schunkert H. (2007). Familial aggregation of left main coronary artery disease and future risk of coronary events in asymptomatic siblings of affected patients. Eur. Heart J.

[5] Fischer M., Broeckel U., Holmer S., Baessler A., Hengstenberg C., Mayer B., Erdmann J., Klein G., Riegger G., Jacob H.J. (2005). Distinct heritable patterns of angiographic coronary artery disease in families with myocardial infarction. Circulation.

[6] Kolovou G., Vasiliadis I., Kolovou V., Karakosta A., Mavrogeni S., Papadopoulou E., Papamentzelopoulos S., Giannakopoulou V., Marvaki A., Degiannis D. (2011). The role of common variants of the cholesteryl ester transfer protein gene in left main coronary artery disease. Lipids Health Dis.

[7] Ross R. (1999). Atherosclerosis—An inflammatory disease. N. Engl. J. Med.

[8] Hansson G.K. (2005). Inflammation, atherosclerosis, and coronary artery disease. N. Engl. J. Med.

[9] Dinarello C.A. (1994). The interleukin-1 family: 10 years of discovery. FASEB J.

[10] Boekholdt S.M., Stroes E.S. (2012). The interleukin-6 pathway and atherosclerosis. Lancet.

[11] Mihara M., Hashizume M., Yoshida H., Suzuki M., Shiina M. (2012). IL-6/IL-6 receptor system and its role in physiological and pathological conditions. Clin. Sci.

[12] Vogiatzi K., Apostolakis S., Voudris V., Thomopoulou S., Kochiadakis G.E., Spandidos D.A. (2008). Interleukin 8 and susceptibility to coronary artery disease: A population genetics perspective. J. Clin. Immunol.

[13] Fichtlscherer S., Breuer S., Heeschen C., Dimmeler S., Zeiher A.M. (2004). Interleukin-10 serum levels and systemic endothelial vasoreactivity in patients with coronary artery disease. J. Am. Coll. Cardiol.

[14] Harismendy O., Notani D., Song X., Rahim N.G., Tanasa B., Heintzman N., Ren B., Fu X.D., Topol E.J., Rosenfeld M.G. (2011). 9p21 DNA variants associated with coronary artery disease impair interferon-gamma signalling response. Nature.

[15] McPherson R., Davies R.W. (2012). Inflammation and coronary artery disease: insights from genetic studies. Can. J. Cardiol.

[16] Deloukas P., Kanoni S., Willenborg C., Farrall M., Assimes T.L., Thompson J.R., Ingelsson E., Saleheen D., Erdmann J., Goldstein B.A. (2013). Large-scale association analysis identifies new risk loci for coronary artery disease. Nat. Genet.

[17] Patel M.R., Dehmer G.J., Hirshfeld J.W., Smith P.K., Spertus J.A., ACCF/SCAI/STS/AATS/AHA/ASNC/HFSA/SCCT 2012 (2012). Appropriate use criteria for coronary revascularization focused update: A report of the American College of Cardiology Foundation Appropriate Use Criteria Task Force, Society for Cardiovascular Angiography and Interventions, Society of Thoracic Surgeons, American Association for Thoracic Surgery, American Heart Association, American Society of Nuclear Cardiology, and the Society of Cardiovascular Computed Tomography. J. Am. Coll. Cardiol.

[18] Tomic V., Russwurm S., Moller E., Claus R.A., Blaess M., Brunkhorst F., Bruegel M., Bode K., Bloos F., Wippermann J. (2005). Transcriptomic and proteomic patterns of systemic inflammation in on-pump and off-pump coronary artery bypass grafting. Circulation.

[19] Calvano S.E., Xiao W., Richards D.R., Felciano R.M., Baker H.V., Cho R.J., Chen R.O., Brownstein B.H., Cobb J.P., Tschoeke S.K. (2005). A network-based analysis of systemic inflammation in humans. Nature.

[20] Podgoreanu M.V., White W.D., Morris R.W., Mathew J.P., Stafford-Smith M., Welsby I.J., Grocott H.P., Milano C.A., Newman M.F., Schwinn D.A. (2006). Inflammatory gene polymorphisms and risk of postoperative myocardial infarction after cardiac surgery. Circulation.

[21] Yamada Y., Izawa H., Ichihara S., Takatsu F., Ishihara H., Hirayama H., Sone T., Tanaka M., Yokota M. (2002). Prediction of the risk of myocardial infarction from polymorphisms in candidate genes. N. Engl. J. Med.

[22] Schaeffeler E., Zanger U.M., Eichelbaum M., Asante-Poku S., Shin J.G., Schwab M. (2008). Highly multiplexed genotyping of thiopurine *S*-methyltransferase variants using MALD-TOF mass spectrometry: Reliable genotyping in different ethnic groups. Clin. Chem.

[23] Chen Z.J., Zhao H., He L., Shi Y., Qin Y., Shi Y., Li Z., You L., Zhao J., Liu J. (2011). Genome-wide association study identifies susceptibility loci for polycystic ovary syndrome on chromosome 2p16.3, 2p21 and 9q33.3. Nat. Genet.

[24] Yi P., Chen Z., Zhao Y., Guo J., Fu H., Zhou Y., Yu L., Li L. (2009). PCR/LDR/capillary electrophoresis for detection of single-nucleotide differences between fetal and maternal DNA in maternal plasma. Prenat. Diagn.

[25] Capodanno D., Di S.M.E., Seminara D., Caggegi A., Barrano G., Tagliareni F., Dipasqua F., Tamburino C. (2011). Epidemiology and clinical impact of different anatomical phenotypes of the left main coronary artery. Heart Vessels.

[26] Iwasaki K., Matsumoto T., Aono H., Furukawa H., Nagamachi K., Samukawa M. (2010). Distribution of coronary atherosclerosis in patients with coronary artery disease. Heart Vessels.

[27] Papanicolaou D.A., Wilder R.L., Manolagas S.C., Chrousos G.P. (1998). The pathophysiologic roles of interleukin-6 in human disease. Ann. Intern. Med.

[28] Jiang C.Q., Lam T.H., Liu B., Lin J.M., Yue X.J., Jin Y.L., Cheung B.M., Thomas G.N. (2010). Interleukin-6 receptor gene polymorphism modulates interleukin-6 levels and the metabolic syndrome: GBCS-CVD. Obesity.

[29] Hamid Y.H., Urhammer S.A., Jensen D.P., Glumer C., Borch-Johnsen K., Jorgensen T., Hansen T., Pedersen O. (2004). Variation in the interleukin-6 receptor gene associates with type 2 diabetes in Danish whites. Diabetes.

[30] Schnabel R.B., Kerr K.F., Lubitz S.A., Alkylbekova E.L., Marcus G.M., Sinner M.F., Magnani J.W., Wolf P.A., Deo R., Lloyd-Jones D.M. (2011). Large-scale candidate gene analysis in whites and African Americans identifies IL-6R polymorphism in relation to atrial fibrillation: the National Heart, Lung, and Blood Institute’s Candidate Gene Association Resource (CARe) project. Circ. Cardiovasc. Genet.

[31] Sarwar N., Butterworth A.S., Freitag D.F., Gregson J., Willeit P., Gorman D.N., Gao P., Saleheen D., Rendon A., Nelson C.P. (2012). Interleukin-6 receptor pathways in coronary heart disease: A collaborative meta-analysis of 82 studies. Lancet.

[32] Hingorani A.D., Casas J.P. (2012). The interleukin-6 receptor as a target for prevention of coronary heart disease: A mendelian randomisation analysis. Lancet.

